# Tracking Varroa Parasitism Using Handheld Infrared Cameras: Is Eusocial Fever the Key?

**DOI:** 10.3390/insects15090693

**Published:** 2024-09-13

**Authors:** Tamás Sipos, Szilvia Orsi-Gibicsár, Tamás Schieszl, Tamás Donkó, Zsombor Zakk, Sándor Farkas, Antal Binder, Sándor Keszthelyi

**Affiliations:** 1 Department of Agronomy, Institute of Agronomy, Kaposvár Campus, Hungarian University of Agriculture and Life Sciences, S. Guba Str. 40, H-7400 Kaposvár, Hungaryostrinia@gmail.com (S.K.); 2 Department of Physiology and Animal Health, Institute of Physiology and Nutrition, Kaposvár Campus, Hungarian University of Agriculture and Life Sciences, Guba Sándor Str. 40, H-7400 Kaposvár, Hungary; 3 Medicopus Nonprofit Ltd., Guba Sándor Str. 40, H-7400 Kaposvár, Hungary; donko.tamas@sic.medicopus.hu; 4 Department of Nature Conservation Biology, Institute for Wildlife Management and Nature Conservation, Kaposvár Campus, Hungarian University of Agriculture and Life Sciences, Guba Sándor Str. 40, H-7400 Kaposvár, Hungary

**Keywords:** *Apis mellifera*, *Varroosis*, social immunity, infrared thermography

## Abstract

**Simple Summary:**

*Varroa destructor* is a significant global honey bee parasite and the primary threat to bee health. Due to its latent lifestyle, detecting the mite in a brood requires invasive techniques. Enhancing detection methods is critical for advanced research on mite population dynamics, spread, selection efforts, and control methodologies. In this study, we employed infrared thermal imaging, a less-explored technique in apicultural studies, to detect parasitism in *Apis mellifera* broods. Our findings indicate that handheld infrared thermal cameras can generate adequately detailed heat maps of the hive. These maps distinctly separate cells containing honey, pollen, and brood, with stable, reproducible temperature measurements observable in late autumn. Notably, mite parasitism induces a sustained temperature increase in developing honey bee pupae, consistently detected regardless of mite numbers in the cell. This study reveals an advanced thermoregulatory behavior in the honey bee colony, manifesting as a social fever phenomenon. Further research is necessary to explore the health benefits of this behavior for bees and the negative effects on the mite. Our method, combined with the development of AI-based image evaluation software, could provide beekeepers and researchers with a valuable tool for *Varroa* research and bee biological studies.

**Abstract:**

The *Varroa destructor* is the most significant bee parasite and the greatest threat to bee health all around the world. Due to its hidden lifestyle, detection within the brood cell is only possible through invasive techniques. Enhancing detection methods is essential for advancing research on population dynamics, spread, selection efforts, and control methodologies against the mite. In our study, we employed infrared imaging to measure the thermal differences in parasite and intact *Apis mellifera* worker broods. Experiments were conducted over two years at the MATE Kaposvár Campus in Hungary involving five beehives in 2022 and five beehives in 2023. A FLIR E5-XT WIFI handheld infrared camera was used to create a heat map of capped brood frames. Our results indicate that the resolution of these cameras is sufficient to provide detailed IR images of a bee colony, making them suitable to detect temperature differences in intact and *Varroa* parasitized capped brood cells. Mite parasitism causes a time-dependent and sustained temperature increase in developing bee pupae, observable regardless of mite number. Our work demonstrates two different heating patterns: hotspot heating and heating cells that are responsible for the elevated temperature of the Varroa-infested cells as a social fever response by the worker bees. Based on our results, future research combined with AI-based image evaluation software could offer beekeepers and researchers practical and valuable tools for high-throughput, non-invasive *Varroa* detection in the field.

## 1. Introduction

As a prominent species among eusocial insects, the honey bee (*Apis mellifera*, L. 1758) exhibits highly developed climate regulation strategies in every season [1,2]. Maintaining homeostasis is crucial, especially because eggs, larvae, and pupae are highly sensitive to temperature fluctuations. The importance of thermoregulation is exemplified by the amount of energy expended: an average-sized colony consumes approximately 2,000,000 kJ of energy in a year for thermoregulation, representing 40% of the total energy expenditure [3]. We accept the correction. optimal ambient temperature for the colony’s development ranges between 32 °C and 36 °C, ensuring healthy growth across different developmental stages [4,5]. Consequently, thermoregulation accuracy is high in the brood nest but becomes more variable and spans a wider range during periods without brood rearing in the wintertime [5,6]. Eggs and larvae tolerate lower temperatures better compared to pupae enclosed in brood cells [6,7]. Pupae exposed to prolonged temperatures below 32 °C are prone to developmental abnormalities, potentially leading to reduced memory and orientation abilities in worker bees [8]. Similarly, larvae exposed to temperatures below 32 °C for more than thirty minutes face significantly increased risks of pathogenic infections. Adult bees also exhibit behavioral and neurological issues due to substantial temperature fluctuations [8,9].

It is well known that insects with variable body temperatures are not able to heat their own environment and body independently, except during the process of chemoregulation, whereby some insects produce heat by burning chemical compounds through muscle work to increase locomotor and metabolic activity. In honey bee colonies, the preimaginal stages (larvae and free pupae) developing in the brood cells are unable to thermoregulate in the absence of active movement [5,6]. Larvae and preimaginal stages are considered poikilothermic, lacking inherent thermoregulation mechanisms and unable to generate sufficient heat independently for proper development; thus, imago worker bees must ensure thermal stability for them [10,11,12]. Honey bees maintain thermal homeostasis of the colony, and especially of the brood, by heating actively with their wing muscles, clustering for insulation when the external temperature is below 10 °C or cooling by vaporizing water and wing fanning when it is above 32 °C [13,14,15]. A colony, as a superorganism, must be understood as a homeothermic organism which tries to control its core temperature at a constant level [15,16,17]. Among eusocial insects, elevated body temperature or social fever is often considered a form of social immunity, closely intertwined with colony homeostasis and thermoregulation [18,19]. Social immunity encompasses diverse behavioral and organizational mechanisms that eusocial insects employ to defend against parasites and pathogens, thereby safeguarding colony health [19]. This collective effort among individuals possibly aims to constrain pathogen and parasite dissemination [20]. Brood temperatures elevated by approximately 0.03–0.19 °C have been observed in the developmental stages of honey bees parasitized by *Varroa destructor* (Anderson and Trueman 2000); however, the precise underlying mechanisms remain unclear [21].

Enhancing detection methods is critical for advanced research on mite population dynamics, spread, selection efforts, and control methodologies [22,23]. Indirect, non-invasive infrared thermal imaging is not unknown in entomological research. This methodology can provide insights into specific characteristics of poikilothermic organisms such as insects by measuring their surface temperature in a non-invasive manner [24,25]. For instance, it has been used to study chemoregulation-generated temperature changes in certain *Lepidopteran* spp. [26], to analyze the thermal characteristics of social insects like bees and wasps [27], and to investigate the specificity of parasitism phenomena in honey bee colonies [21,28]. Overall, the method is effective for indirectly mapping physiological changes in individual and social insects [21,29]. Consequently, the information obtained can be used to infer direct biological phenomena [21,30,31]. However, the number of research studies utilizing this methodology is limited, and its potential has thus far been only partially exploited.

The aim of our field and laboratory research was to map the temperature patterns observed on the capped brood using an infrared thermal camera (IR). Over the course of our two-year study, we investigated whether physiological changes due to varroosis are related to temperature variations in developing individuals. Ultimately, our work aims to increase understanding of the thermoregulation mechanism of honey bee colonies and explore the possibilities for indirect mapping of *Varroa* mite to lay the foundations for a potential new prodiagnostic method.

## 2. Materials and Methods

### 2.1. Colony Setup

The experiments were carried out in 2022 and 2023. In both years, the experiments were conducted at the same location and with the same settings. For the duration of the experiment, 5 hives were set up each year on the campus of the Hungarian University of Agricultural and Life Sciences in Kaposvár (WGS:X:46.381259, Y:17.82654). The experimental colonies were limited to 8 Nagyboczonádi (Nb)-size frames built to withstand cold in a standard Hungarian vertical-type hive; the same hives were used in each year. In order to avoid the results of the experiment being influenced by varroosis symptoms and, thus, the premature death of the colonies, artificial swarms were created. The colonies were established on 7 June 2022 for the 2022 year and on 7 June 2023 for the 2023 year; the experiments were carried out using the same technique. The artificial swarms contained 1.5 kg of worker bees and a one-year-old Carniolan (*Apis mellifera carnica*) queen. The bees were installed on 8 NB frames with 6 kg of honey in identical hives, and a pollen supply was added. The colonies received 0.5 L of 50% of sucrose solution for two weeks until the nucleus colonies were fully established. The queens of the colonies were from non-varroa-tolerant lines. Our objective was to mimic the natural mite infestation process by sublimating 2 g of oxalic acid (99.5% purity) twice, thus delaying the increase of phoretic *Varroa* mites by 5 and 8 days, respectively, after establishing the colonies. For the duration of the infrared thermography measurements, the colonies were nearly identically strong, and they were extended to 8 active frames, with an almost equal number of brood frames (2–3). To ensure that heat loss in the hives did not affect the results of the experiment, the top of the frames was fully covered with 400 µm thick polyethylene foil for agriculture and the top of the hives was insulated with a 4 cm thick expanded polystyrene thermal insulation board.

### 2.2. Brood Temperature-Monitoring Thermal Imaging Studies

Thermal imaging was conducted for five days constantly prior to dissection at consistent intervals in the afternoon between 12:00 and 16:00 for both years. The experiments were carried out from October 10 to 15 in 2022 and 2023 under similar weather conditions. The temperature was 18–23 °C in both years during the afternoons when the thermal imaging was carried out. The images were captured using a FLIR E5-XT WIFI handheld infrared thermal imager with a resolution of 160 × 120 pixels, a frame rate of 9 Hz, an accuracy of 0.1 °C, and a 0.95 emissivity rate. All images of the specimens were utilized as the basis for the experiment, with the thermal images taken from a uniform distance of 50 cm. To ensure that the brood surfaces did not cool within 30 s of removal, the imaging was performed in the back of a van: this was an enclosed space shielded from direct sunlight, maintained at a temperature of 23 °C. Prior to capturing the thermal images, the frames were shaken to remove imago worker bees, and the images were then captured.

The thermal images were analyzed using Teledyne FLIR Thermal Studio. In order to compare the infrared images of the same brood frame in the function of time, the color scale of the thermal images was converted to grayscale coloring by FLIR Thermal Studio. The converted grayscale images’ pixel intensity histograms were analyzed with the help of GIMP 2.10.32 software. All five thermal images captured at different time points (from 10 to 15 October) were used to assess the thermal imaging patterns of the colonies.

To examine different developmental stages of brood and intact and infested cell temperatures, a polyline tool was employed to measure the exact hexagonal areas bounded by the brood cells, each containing an average of 38 ± 4 pixels. The average temperature values of the hexagon-shaped areas were recorded. Conversely, evaluation of the effect of mites on the temperature of the capped cells was based on thermal images captured on the 5th day (15 October) of each year. The 21 brood frames’ thermal values were analyzed retrospectively after dissection, measuring the parasitized cells and adjacent intact brood cells at the same stage of development. Temperature values were determined for a total of 1005 parasitized and 1005 intact individuals of mixed age composition, selected according to specified criteria, over the two years. These individuals’ data were also used to calculate the statistical differences in the developmental stages.

### 2.3. Dissection of the Capped Brood

After the five thermal images of each brood frame were captured over five days for each hive, the frames were removed from the hive to ensure that the location of the mites and the developmental state of the specimens remained similar compared to the thermal images. The frames were then stored at −20 °C until dissections were started. To allow for the dissection of the brood cells, the frames were placed in an incubator at 30 °C for 30 min to ensure proper thawing. During the dissections, the developmental status of the bees was determined based on the work of Rembold et al., 1980 [32], along with the presence of parasitization. All the capped broods from both sides of the frames were dissected. For the parasitized cells, the number of mites was recorded, and the location of the cell was documented on the pre-captured photographs to facilitate retrospective temperature determination. Dissections were conducted using a laboratory microchip under a stereomicroscope [33]. For each brood cell, the stage of development of the individuals was noted on the pre-captured photographs, and the number and the developmental stages of the parasites were recorded.

### 2.4. Data Analysis

To test the normal distribution of the data, a Shapiro–Wilk normality test was performed (*p* ≤ 0.05). To establish the consistency of the heating pattern of the brood nest, the identity of the thermal images captured at five different time points prior to the experiment was tested, and the pixel intensity histograms were compared using Pearson correlation analysis. To evaluate the differences between developmental stages, paired *t*-test was used (*n* = 100) (*p* ≤ 0.05). To calculate the thermal differences in various pupal stages (*n* = 200), one-way ANOVA was carried out. To determine the effect of the *Varroa* mite on temperature, a one-way ANOVA was performed for intact and parasitized individuals (*n* = 1005) (*p* ≤ 0.05). The effect of the number of mites on temperature difference was tested using a one-way analysis of variance (*p* ≤ 0.05). Temperature distribution on the surface of parasitized brood cells parasitized in the case of different numbers of *Varroa* mites was calculated by one-way ANOVA (*p* ≤ 0.05) (*n* = 508). Statistical tests were conducted using the R-4.2.1 and Microsoft Excel software packages.

## 3. Results

### 3.1. Abundance of Varroa Destructor in the Brood

**Figure 1:**
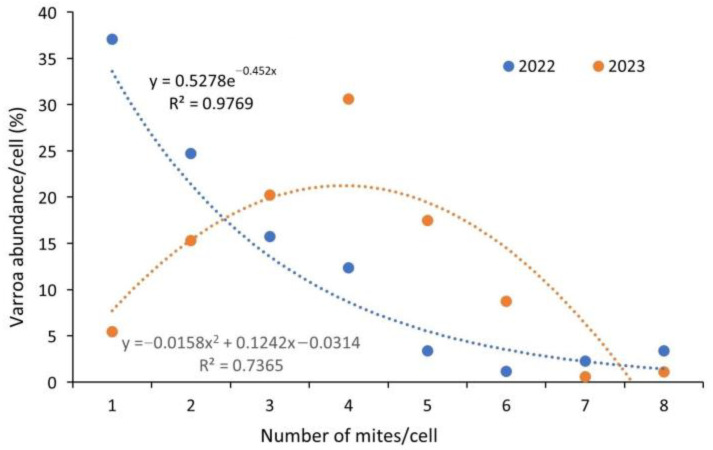
Plots of the relative abundance of mites in dissected brood cells over two years revealed differing trends.

During the experiments in 2022, we dissected 10 NB-size frames containing 8887 capped worker broods; during the work in 2023, we dissected 9256 cells on 11 NB frames. The colonies’ brood, on average, were 5.653% parasitized with Varroa mites in 2022 compared to 5.435% in 2023. The infestation level of the experimental colonies’ brood varied, ranging from 3.117% to 12.936% in the two years. Dissection of 1.005 mixed-age worker cells infested by Varroa mites demonstrated that the number of mites in a brood cell can vary from 1 parasite to 13 parasites. If we examine the absolute prevalence of mite numbers in a cell, we find that the most common is a single parasite in the year 2022, while with an increasing number of mites in a cell, a power-type decreasing trend is demonstrated. An exponential decay curve describing the variation in the abundance of mite numbers in a cell shows a tight correlation, which is well represented by the value of the coefficient of determination (R^2^ = 0.98). The population dynamics of the mite were considerably different in 2023: the most frequent prevalence was four mites in a cell, and the distribution of mites followed a parabolic curve, which is shown in Figure 1.

In 2022, mite abundance followed an exponential trend line. In contrast, the evolution of mite abundance in the 2023 sampling was best described by a second-degree polynomial equation. The most significant difference observed was a nearly 30% higher prevalence of brood cells containing a single mite in 2022 compared to 2023.

### 3.2. Comparison of Thermal Images of the Brood Nest

**Figure 2:**
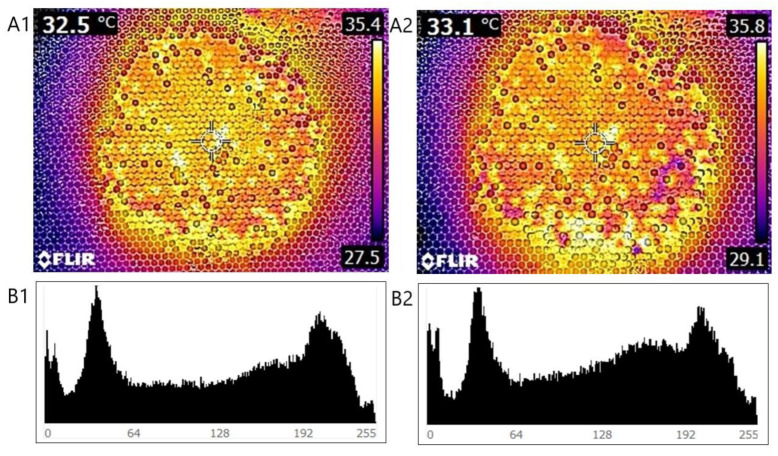
Thermal images of the same hive and frame captured at different time points ((**A1**) 23.10.14; (**A2**) 23.10.15), along with pixel intensity histograms (**B1**,**B2**) generated from digital image processing.

As a result of testing the temperature values, it was observed that the temperatures of the different types of cells are clearly distinguished and show significant variation. It can be concluded that in the late autumn period, active heating is mainly concentrated in the brood chamber. The areas with higher temperatures are generally clustered around a well-defined brood area. A similar patchy, nodular pattern can be observed for the areas that can be characterized by lower temperatures, such as the honey-containing and pollen-containing cells. According to correlation analysis of the pixel-based intensity parameters extracted from the histograms of the images shown in Figure 2, it was found that between the pixel intensity parameters, there are significant similarities between the identities of the thermal images captured on 2 subsequent days (r = 0.999). The evolution of the temperatures of the brood chamber as a function of time shows a significant similarity, which indicates stable temperature-control activity of the workers in the areas concerned, linked to the brood areas. It can be clearly seen from the images that the workers produce most of the thermal energy for the brood (red, orange, and yellow colorization area in Figure 2), whereas there was no active heat production in other areas in the honey-storing cells nor in the pollen-storing cells (blue colorization area in Figure 2).

**Figure 3:**
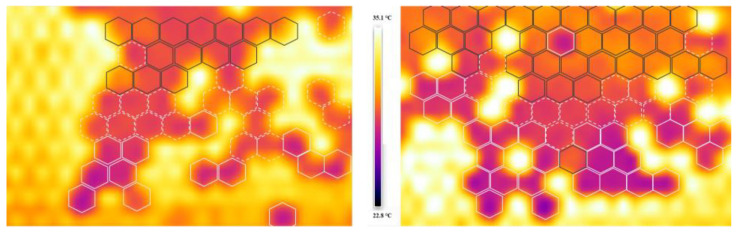
Thermal map of brood with capped larvae (marked white hexagons), pre-pupae (scattered white hexagons), and older pupae (black hexagons).

The brood area was determined to comprise two well-separated parts; these can be seen on the thermal images in Figure 3. The temperature differences are clearly visible; larvae and early-stage pupae (marked with white and white-striped hexagons) exhibit significantly lower temperatures compared to older pupae (marked with black hexagons). Based on the thermal values of the capped brood, the sealed larvae (LS), prepupa (Pp), (mean temperature value: 28.96 ± 0.70 °C) and pupal stages were well separated, which was statistically confirmed (df = 87; *p* < 0.001). The larval and the prepupa stages could be separated on the edge of the brood and in the middle of the brood. The pupae also could be separated based on the capping temperatures that were confirmed by one-way ANOVA statistical probing (df = 5; F = 4.051375; *p* = 0.001451). The temperature differences were calculated for the different developmental stages. The mean values of different developmental stages of pupae were the following: white-eyed (29.96 ± 0.22 °C); pink-eyed (30.21 ± 0.60 °C); red-eyed (30.27 ± 0.80 °C); dark-brown-eyed and dark-brown-eyed with a lightly pigmented thorax (30.58 ± 0.88 °C); dark-brown-eyed with a medium-dark thorax (30.52 ± 0.68 °C); dark-brown-eyed with a dark thorax (30 ± 0.81 °C).

### 3.3. Relationship between Temperature Rise and Varroa Mite Presence

**Figure 4:**
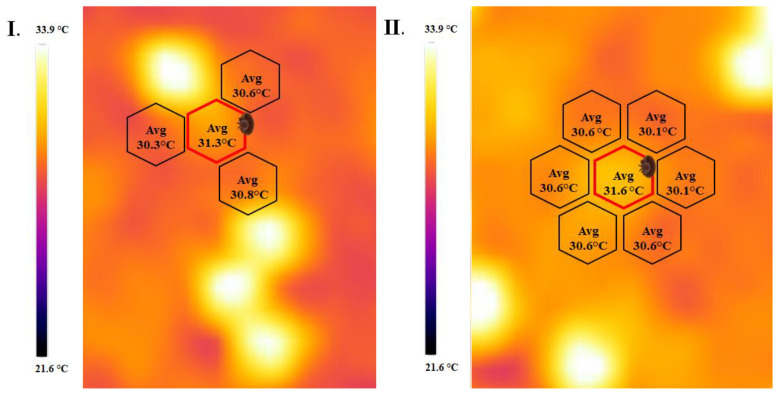
The two most common forms of elevated temperatures caused by Varroa mite. (**I.**) parasitized cell next to a heating cell; (**II.**) A hotspot pattern with the mite-infested brood cell in the center; black hexagon indicates intact; red hexagon indicates parasitized cells.

The results of infrared (IR) thermal imaging of sealed brood cells of worker bees showed that higher temperatures could be detected on the surface on the wax capping of the developing pupae infested by Varroa mites. The differences can be seen in the thermal images in Figure 4. One of the main findings of the research is that two different heating patterns were recognized on the thermal images, both of which were responsible for the elevated temperature of the parasitized cells. The most common heating pattern is the heating cell, as shown on the left pane in Figure 4(I.). The parasitized cell next to a heating cell, indicated by the hexagon marked with the mite, shows a higher temperature compared to the neighboring cells. A hotspot-elevated temperature pattern can be observed in the right pane in Figure 4(II.). The mite-infested brood cell in the center shows a similar tendency: on average, this cell is 0.65 °C warmer than the neighboring cells. The same tendency was measured during the two years when we compared intact individuals at the same stage of development that were neighboring parasitized individuals. The average temperature of infested individuals was 30.78 ± 0.09 °C, approximately 0.82 °C higher than that of intact worker cells (29.96 ± 0.08 °C) in 2022. The 2023 studies similarly confirmed the fact that parasitized cells had a higher temperature (30.62 ± 0.14 °C) than adjacent, intact individuals (29.86 ± 0.08 °C), averaging 0.76 °C higher. One-way analysis of variance confirmed in both experimental years that brood cells with parasite development alongside individuals of different preimaginal stages had statistically verifiably higher temperatures than those without mites (df = 1; F = 118.76; *p* < 0.001).

**Figure 5:**
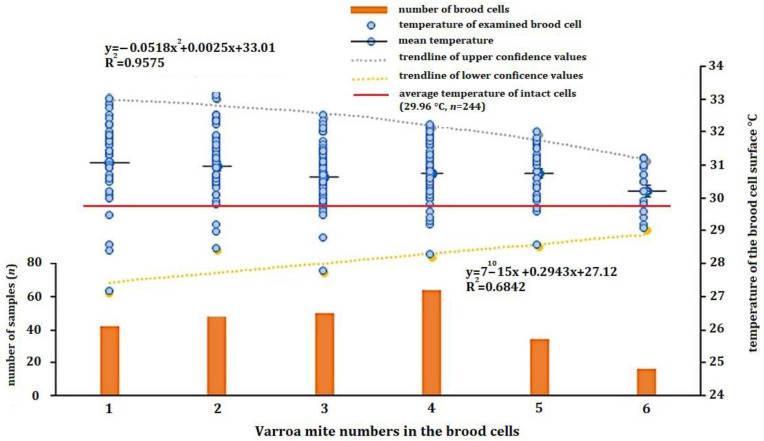
Temperature distribution of the surface of intact and parasitized brood cells as a function of different numbers of Varroa mites (*n* = 508).

The temperature values measured on the cap surface, with different numbers of mites in the cell, are shown in Figure 5. Brood cells with a high number of mites/cell (7 (*n* = 3), 8 (*n* = 5) and 13 (*n* = 1)) were excluded from the statistical analyses because of the low number of elements. The single-factor analysis of variance confirmed that there was a statistically valid correlation between the number of mites in a brood cell and the average value of the surface temperatures of the parasitized cells (df = 1; F = 57,919.99; *p* = 0.001). As the number of mites increases, the confidence interval of the surface temperatures becomes narrower, described by a polynomial decrease in the trend lines drawn at the upper and lower limits of confidence. This verifiable correlation is shown by the result of the statistical analysis of the confidence intervals between the minimum and maximum temperature values measured on brood cells loaded with different numbers of mites. However, it can be observed that the evolution of the mean temperature values with an increasing number of mites per brood cell is stable within a range of 30.0–30.5 °C, representing the mean value. The mean value of the intact cells was 29.98 °C. At a low level of parasitation (one mite/cell: 32.9–27.1 °C; two mites/cell: 33.0–28.4 °C), the mean surface temperatures are within a wide range, whereas at a Varroa load of three mites and above (three mites/cell: 32.4–27.7 °C; four mites/cell: 32.1–29.1 °C; five mites/cell: 31.9–28.5 °C; six mites/cell: 31.1–29 °C), the surface temperatures are continuously narrowing.

## 4. Discussion

Monitoring the abundance of *Varroa* mites in different countries and seasons is important for the study of *Varroa* population dynamics [34]. The rapid spread of *Varroa* mites is illustrated by this two-year study: swarms can reach a high mite count in the brood 4 months after establishment. This increase can range from 3.117% to 12.936% [35]. In our experiment, the high infestation levels in both years could be explained by the high bee density in Hungary, which plays a primary role in the distribution of the mite through the drifting of worker bees [36,37,38]. Our observations on mite population dynamics provided us with indicators that were consistent with those found in the literature, both for larval and pupal stages [39]. The results of the 2022 sampling were atypical for the population dynamics of the mite compared to the 2023 sampling, which represented a normal population dynamics trend [39]. We can confirm that the number of parasites per worker brood cell at the pupal stage was low in 2022: it was predominantly between one and two mites/cell for the pupal stages. Few of the samples analyzed had more than four mites/cell. In contrast, when analyzing samples from 2023, in terms of the mixed-age composition of the dissected pupas, the most dominant number of mites in the cell was four. The differences in the two-year study can be attributed to seasonal patterns of varying nutrition throughout the bee pasture. Thus, colonies’ performance can be influenced by weather conditions [39,40].

Our studies have shown that temperature analysis based on infrared imaging can be a suitable method for monitoring temperature changes in a colony of honey bees, even over five days, as continuous experiments show (r = 0.999) [41]. Furthermore, it can be used to detect the presence of possible *Varroa* mite parasitism in brood cells and to isolate the different honey bee developmental stages. Our study employs a new approach to investigate the temperature dynamics of the brood and hive. Since removing frames from the hive affects temperatures, our measurements were conducted within a specified time interval under controlled environmental conditions. This investigation is grounded in the principles of thermodynamics, allowing us to infer the thermal energy of the bodies [42,43]. By ensuring that the measured objects are composed of the same material, we maintain the comparability of the temperature values. In our study, we observed that the larval and pupal stages of honey bees develop at different temperatures. This variation can be attributed to the distinct temperature requirements specific to each developmental stage [21,44], but further detailed research is needed with higher-resolution infrared cameras. Our results confirmed that the mite-parasitized brood has, on average, a 0.79 °C higher temperature over the two-year period, which was statistically demonstrable. The results of Bauer et al. [21] indicated that the increased values could be explained by elevated metabolic processes induced by mite-transmitted bee viruses. In our opinion, the explanation for this phenomenon can be traced back to the intense social behavior of imagoes. The poikilothermic nature of arthropods explains our position, as developmental forms have an environment-dependent and variable body temperature that is independent of exposure to pathogens. Honey bee workers act as homeotherms, according to this point of view, to establish the optimal temperature and humidity for the developing pupae. The concentrated heating of a single cell in a capped brood can be explained by the so-called hotspot phenomenon [15], whereby worker bees release the thermal energy produced by pressing their thorax onto the brood surface in a concentrated manner on a single cell, especially the *Varroa*-parasitized cells. Another concentrated heating technique that plays a role in social fever is the so-called empty hot brood cells concept [45]; this can guarantee the local heating of infested individuals. The possible explanation for social fever is that workers sense the presence of mites in the cell. The workers try to shift the ambient temperature of the parasitic foreign organism out of the optimal range by active heating, thus worsening the biological indicators and living conditions of the parasitic organism [6,20,21]. An interesting observation of our study is that the variance in the temperature values measured on parasitized brood cells varies strongly depending on the number of parasites within the cell. For the parasite number in a cell, higher temperature values with less variability were obtained from our study for a higher number of infested cells (three mites/cell). The narrowing of the temperature confidence intervals with increasing parasite counts occurred in parallel with parasite detection of the infested individuals by workers, as in the case of hygienic behavior. The difference in variance is explained by the fact that workers are expected to detect parasitized cells with a higher success rate; also, the damage to the brood increases with the number of mites. The damage produces various volatile components [46,47] that can trigger social behavior and, thus, social fever. In contrast, brood cells with a low parasite load are less likely to be detected, maybe because they are in the beginning stages of the varroosis infestation. This fact explains the lower temperature variance in brood cells with a higher parasite count and the increasingly narrow range of temperature values on the surface.

Overall, the primary finding of our experimental work is that non-invasive thermal image analysis, utilizing a handheld thermal camera, can effectively locate *Varroa destructor* in a capped honey bee brood. Following further studies, refinement of this method could lead to a widely applicable technique for non-invasive, high-throughput detection of varroosis under field conditions. A significant advantage of this method is its potential for use by beekeepers without requiring specialized knowledge. Notably, the sensitivity of the thermal imaging device used in our study is comparable to that of sensors in most modern high-end smartphones. Consequently, accessible devices could be employed to optimize the timing of mite control in apiaries, aligning with integrated pest management criteria. Additionally, our method could expedite and validate selection efforts for desirable traits in bee breeding, such as *Varroa* sensitive hygiene (VSH) and suppressed mite reproduction (SMR), following further large-scale testing. The drawbacks of this method include the need for the development of AI-based image evaluation software which can reliably identify the target objects after a learning process for the temperature pattern of each brood comb. To isolate infested brood cells, the program must incorporate prior knowledge such as the temperature thresholds of intact and invested cells and specific temperature patterns of the brood, which can be effectively handled by a deep learning model based on artificial intelligence. To ensure the accuracy of this determination, it is essential to consider a combination of factors, including heating patterns and potential measurement errors. Additionally, the method needs to be tested during hot periods in summer to determine the temperature of specific brood cells in bees without active heating. This will provide deeper insights into the biological mechanisms underlying social fever in bees and will help to evaluate the detection method.

## 5. Conclusions

Our study has demonstrated that a handheld infrared thermal camera, which is easy and simple to use in field conditions, can effectively provide indirect visualization of changes in bee life activities. The thermal energy released by the active muscular activity of worker bees significantly influences the temperature of the brood cells, which is demonstrably related to the parasitization of the brood cells by *Varroa* mites. In the future, this method could be suitable for non-invasive visualization of the spread and presence of *Varroa* mites. The direct outcome of our work is the ability to detect varroosis symptoms without the need for direct dissection of brood cells. For practitioners, this methodology offers a practical opportunity to obtain rapid, non-invasive, and indirect information to assess the number of varroa mites and the health status of bee colonies. However, the method limitations include the costly tool requirements and the lack of a fully developed software support interface, which is essential for optimal performance. Further studies are needed for the summer period, during which bees actively cool the hive to maintain an optimal temperature range. With further improvements in the technique and additional validation trials, this method could become a valuable prognostic tool for large-scale apiaries. The results will contribute to the successful prediction and control of varroosis and may provide a non-invasive, time-efficient tool for varroa research and bee breeding selection works.

## Data Availability

The raw data supporting the conclusions of this article will be made available by the authors on request.

## References

[1] Cook C.N., Kaspar R.E., Flaxman S.M., Breed M.D. (2016). Rapidly changing environment modulates the thermoregulatory fanning response in honeybee groups. Anim. Behav..

[2] Jones J.C., Benjamin P.O. (2006). Nest thermoregulation in social insects. Adv. Insect Physiol..

[3] Tautz J., Maier S., Groh C., Rössler W., Brockmann A. (2003). Behavioral performance in adult honey bees is influenced by the temperature experienced during their pupal development. Proc. Natl. Acad. Sci. USA.

[4] Petz M., Stabentheiner A., Crailsheim K. (2004). Respiration of individual honeybee larvae in relation to age and ambient temperature. J. Comp. Physiol. B.

[5] Stabentheiner A., Kovac H., Brodschneider R. (2010). Honeybee colony thermoregulation–regulatory mechanisms and contribution of individuals in dependence on age, location and thermal stress. PLoS ONE.

[6] Seeley T.D. (2009). The Wisdom of the Hive: The Social Physiology of Honey Bee Colonies.

[7] Starks P.T., Gilley D.C. (1999). Heat shielding: A novel method of colonial thermoregulation in honey bees. Naturwissenschaften.

[8] Groh C., Tautz J., Rössler W. (2004). Synaptic organization in the adult honey bee brain is influenced by brood-temperature control during pupal development. Proc. Natl. Acad. Sci. USA.

[9] Abou-Shaara H.F., Owayss A.A., Ibrahim Y.Y., Basuny N.K. (2017). A review of impacts of temperature and relative humidity on various activities of honey bees. Insectes Sociaux.

[10] Stabentheiner A., Kovac H., Mandl M., Käfer H. (2021). Coping with the cold and fighting the heat: Thermal homeostasis of a superorganism, the honeybee colony. J. Comp. Physiol. A.

[11] Moritz R., Southwick E.E. (2012). Bees as Superorganisms: An Evolutionary Reality.

[12] Lindauer M. (1955). The water economy and temperature regulation of the honeybee colony. Bee World.

[13] Heinrich B. (1981). The mechanisms and energetics of honeybee swarm temperature regulation. J. Exp. Biol..

[14] Southwick E.E., Heldmaier G. (1987). Temperature control in honey bee colonies. Bioscience.

[15] Bujok B., Kleinhenz M., Fuchs S., Tautz J. (2002). Hot spots in the bee hive. Naturwissenschaften.

[16] Southwick E.E. (1982). Metabolic energy of intact honey bee colonies. Comp Biochem. Physiol. A.

[17] Ivanov K.P. (2006). The development of the concepts of homeothermy and thermoregulation. J. Therm. Biol..

[18] Starks P.T., Blackie C.A., Seeley T. (2000). Fever in honeybee colonies. Naturwissenschaften.

[19] De Roode J.C., Lefèvre T. (2012). Behavioral immunity in insects. Insects.

[20] Goblirsch M., Warner J.F., Sommerfeldt B.A., Spivak M. (2020). Social fever or general immune response? Revisiting an example of social immunity in honey bees. Insects.

[21] Bauer D., Wegener J., Bienefeld K. (2018). Recognition of mite-infested brood by honeybee (*Apis mellifera*) workers may involve thermal sensing. J. Therm. Biol..

[22] Sipos T., Donkó T., Jócsák I., Keszthelyi S. (2021). Study of Morphological Features in Pre-Imaginal Honey Bee Impaired by *Varroa destructor* by Means of Computer Tomography. Insects.

[23] Sipos T., Glavák C., Turbók J., Somfalvi-Tóth K., Donkó T., Keszthelyi S. (2024). Analysis of X-ray irradiation effects on the mortality values and hemolymph immune cell composition of *Apis mellifera* and its parasite, *Varroa destructor*. J. Invertebr. Pathol..

[24] Campbell A.L., Naik R.R., Sowards L., Stone M.O. (2002). Biological infrared imaging and sensing. Micron.

[25] Harrap M.J., Hempel de Ibarra N., Whitney H.M., Rands S.A. (2018). Reporting of thermography parameters in biology: A systematic review of thermal imaging literature. R. Soc. Open Sci..

[26] Kim J. (2022). Skin temperature characteristics of the ligustrum moth (*Brahmaea certhia*) and the hawk moth (*Theretra oldenlandiae*) using IR camera. J. Mech. Sci. Technol..

[27] Stabentheiner A., Kovac H., Hetz S.K., Käfer H., Stabentheiner G. (2012). Assessing honeybee and wasp thermoregulation and energetics—New insights by combination of flow-through respirometry with infrared thermography. Thermochim. Acta.

[28] Bjerge K., Frigaard C.E., Mikkelsen P.H., Nielsen T.H., Misbih M., Kryger P. (2019). A computer vision system to monitor the infestation level of *Varroa destructor* in a honeybee colony. Comput. Electron. Agric..

[29] Johnson A.P., Wallman J.F. (2014). Infrared imaging as a non-invasive tool for documenting maggot mass temperatures. Aust. J. Forensic Sci..

[30] Hunt V.L., Lock G.D., Pickering S.G., Charnley A.K. (2011). Application of infrared thermography to the study of behavioural fever in the desert locust. J. Therm. Biol..

[31] Klein B.A., Stiegler M., Klein A., Tautz J. (2014). Mapping sleeping bees within their nest: Spatial and temporal analysis of worker honey bee sleep. PLoS ONE.

[32] Rembold H., Kremer J.P., Ulrich G.M. (1980). Characterization of postembryonic developmental stages of the female castes of the honey bee, *Apis mellifera* L.. Apidologie.

[33] Dietemann V., Nazzi F., Martin S.J., Anderson D.L., Locke B., Delaplane K.S., Wauquiez Q., Tannahill C., Frey E., Ziegelmann B. (2013). Standard methods for varroa research. J. Apic. Res..

[34] Messan K., Messan M.R., Chen J., DeGrandi-Hoffman G., Kang Y. (2021). Population dynamics of *Varroa* mite and honeybee: Effects of parasitism with age structure and seasonality. Ecol. Model..

[35] Giacobino A., Miotti C., Molineri A., Orellano E., Signorini M., Pacini A. (2023). *Varroa destructor* re-invasion dynamics during autumn and winter in *Apis mellifera* colonies from a temperate climate. J. Invertebr. Pathol..

[36] Farkas Á., Zajácz E. (2007). Nectar production for the Hungarian honey industry. Eur. J. Plant Sci. Biotechnol..

[37] Peck D.T., Seeley T.D. (2019). Mite bombs or robber lures? The roles of drifting and robbing in *Varroa destructor* transmission from collapsing honey bee colonies to their neighbors. PLoS ONE.

[38] Bava R., Castagna F., Palma E., Ceniti C., Millea M., Lupia C., Britti D., Musella V. (2023). Prevalence of *Varroa destructor* in honeybee (*Apis mellifera*) farms and varroosis control practices in Southern Italy. Microorganisms.

[39] Rosenkranz P., Aumeier P., Ziegelmann B. (2010). Biology and control of *Varroa destructor*. J. Invertebr. Pathol..

[40] Aronstein K.A., Saldivar E., Vega R., Westmiller S., Douglas A.E. (2012). How Varroa parasitism affects the immunological and nutritional status of the honey bee, Apis mellifera. Insects.

[41] Klein B.A., Busby M.K. (2020). Slumber in a cell: Honeycomb used by honey bees for food, brood, heatin and sleeping. PeerJ.

[42] Fermi E. (2012). Thermodynamics.

[43] Lee J.H., Ramamurthi K. (2022). Fundamentals of Thermodynamics.

[44] Czekońska K., Tofilski A. (2020). Body mass of honey bee drones developing in constant and in changing temperatures. Apidologie.

[45] Kleinhenz M., Bujok B., Fuchs S., Tautz J. (2003). Hot bees in empty broodnest cells: Heating from within. J. Exp. Biol..

[46] Martin C., Provost E., Bagnères A.G., Roux M., Clément J.L., Le Conte Y. (2002). Potential mechanism for detection by *Apis mellifera* of the parasitic mite *Varroa destructor* inside sealed brood cells. Physiol. Entomol..

[47] Light M., Shutler D., Cutler G.C., Hillier N.K. (2020). *Varroa destructor* mite electrophysiological responses to honey bee (*Apis mellifera*) colony volatiles. Exp. Appl. Acarol..

